# Behind the Uniform: Prevalence and Determinants of Alcohol Use Disorder in the Tunisian Military

**DOI:** 10.1192/j.eurpsy.2026.10872

**Published:** 2026-07-31

**Authors:** A. Kammoun, L. S. Chaibi, A. Aissa, A. Oumaya

**Affiliations:** 1 Psychiatry; 2 Hopital Militaire Principal d’Instruction de Tunis; 3 Hopital Razi, Tunis, Tunisia

## Abstract

**Introduction:**

Alcohol Use Disorder (AUD) is disproportionately prevalent in military populations, fueled by combat exposure, psychological stress, and early-life trauma. Strongly linked to PTSD and other psychiatric comorbidities, AUD undermines both health and operational performance.

**Objectives:**

The present study aims to explore the prevalence and determinants of Alcohol Use Disorder (AUD) in Tunisian military personnel, focusing on the roles of childhood trauma, combat exposure, and PTSD.

**Methods:**

A cross-sectional descriptive and analytical study was conducted from September to December 2024 at the outpatient military psychiatry unit of the Principal Military Teaching Hospital in Tunis. The study included Tunisian military personnel followed for a psychiatric disorder for more than six months, who provided written informed consent. Individuals with psychotic disorders or incomplete questionnaires were excluded. Data were collected using sociodemographic forms and validated instruments, including the AUDIT for alcohol use, the Childhood Trauma Questionnaire (CTQ), the PTSD Checklist for DSM-5 (PCL-5), and the PHQ-9 for depression. Statistical analyses included t-tests, ANOVA, and chi-square or Fisher’s exact tests, with significance set at p < 0.05.

**Results:**

The study included 60 Tunisian military personnel (58 men, 2 women) with a mean age of 33.6 ± 0.8 years. About half were married (51.7%), and 35% had a university-level education, while most had completed secondary school. Alcohol Use Disorder (AUD) was more prevalent in personnel from the navy and army compared to the air force.

Analytical results showed significant associations between AUD and several determinants. Combat exposure was strongly correlated with higher alcohol consumption (p < 0.001). Childhood trauma, assessed by the CTQ, was significantly higher in participants with AUD (p = 0.003), with physical abuse showing a notable link (p = 0.016). PTSD severity, measured by the PCL-5, was also significantly associated with AUD (p < 0.001). Finally, depressive symptoms were correlated with alcohol use, indicating that higher depression severity corresponded to greater alcohol consumption

**Conclusions:**

Alcohol Use Disorder affects 60% of Tunisian military personnel followed in psychiatry, with combat exposure and PTSD emerging as key determinants. These findings highlight the critical impact of operational stress on alcohol use and the urgent need for targeted prevention and intervention strategies. This study provides the first insight into AUD in Tunisia’s military, laying the groundwork for future research and tailored mental health programs.

**Disclosure of Interest:**

None Declared

